# Activation of Eosinophils Interacting with Dermal Fibroblasts by Pruritogenic Cytokine IL-31 and Alarmin IL-33: Implications in Atopic Dermatitis

**DOI:** 10.1371/journal.pone.0029815

**Published:** 2012-01-17

**Authors:** Chun-Kwok Wong, Karen Ming-Lam Leung, Huai-Na Qiu, Joyce Yin-Sau Chow, Angela On Kei Choi, Christopher Wai-Kei Lam

**Affiliations:** 1 Department of Chemical Pathology, The Chinese University of Hong Kong, Prince of Wales Hospital, Hong Kong, Special Administrative Region, People's Republic of China; 2 Macau Institute for Applied Research in Medicine and Health, Macau University of Science and Technology, Taipa, Macau; Centre de Recherche Public de la Santé (CRP-Santé), Luxembourg

## Abstract

**Background:**

IL-31 is a pruritogenic cytokine, and IL-33 is an alarmin for damaging inflammation. They together relate to the pathogenesis of atopic dermatitis (AD). Eosinophil infiltration into the inner dermal compartment is a predominant pathological feature of AD. We herein investigated the *in vitro* inflammatory effects of IL-31 and IL-33 on the activation of human eosinophils and dermal fibroblasts.

**Methodology/Principal Findings:**

Receptors, adhesion molecules and signaling molecules were assessed by Western blot or flow cytometry. Chemokines and cytokine were quantitated by multiplex assay. Functional IL-31 receptor component IL-31RA, OSMR-β and IL-33 receptor component ST2 were constitutively expressed on the surface of eosinophils. Co-culture of eosinophils and fibroblasts significantly induced pro-inflammatory cytokine IL-6 and AD-related chemokines CXCL1, CXCL10, CCL2 and CCL5. Such inductions were further enhanced with IL-31 and IL-33 stimulation. IL-31 and IL-33 could significantly provoke the release of CXCL8 from eosinophils and fibroblasts, respectively, which was further enhanced upon co-culture. In co-culture, eosinophils and fibroblasts were the main source for the release of CCL5, and IL-6, CXCL1, CXCL8, CXCL10 and CCL2, respectively. Direct interaction between eosinophils and fibroblasts was required for CXCL1, CXCL10, CXCL8 and CCL5 release. Cell surface expression of intercellular adhesion molecule-1 on eosinophils and fibroblasts was up-regulated in co-culture upon IL-31 and IL-33 stimulation. The interaction between eosinophils and fibroblasts under IL-31 and IL-33 stimulation differentially activated extracellular signal-regulated kinase, c-Jun N-terminal kinase, p38 mitogen-activated protein kinase, nuclear factor-κB and phosphatidylinositol 3-kinase–Akt pathways. Using specific signaling molecule inhibitors, the differential induction of IL-31 and IL-33-mediated release of cytokines and chemokines such as IL-6 and CXCL8 from co-culture should be related to their distinct activation profile of intracellular signaling pathways.

**Conclusions/Significance:**

The above findings suggest a crucial immunopathological role of IL-31 and IL-33 in AD through the activation of eosinophils-fibroblasts interaction via differential intracellular signaling mechanisms.

## Introduction

Interleukin (IL)-31 is a novel T helper (Th) type 2 cytokine which is mainly produced by the CD45RO+ cutaneous lymphocyte antigen (CLA)+ T lymphocytes [1]. Over-expressed IL-31 could induce pruritus and skin dermatitis resembling human atopic dermatitis (AD) in mice [2], [3]. Anti-IL-31-antibody could ameliorate the scratching behavior in murine model of AD [4]. AD is a pruritic and chronically relapsing inflammatory skin disease with increasing prevalence, that exhibits adverse impact on the quality of life [5], [6]. Plasma IL-31 concentration was found to be significantly elevated in AD patients compared to healthy individuals [7], and correlated positively with disease severity [8]. In the inflammatory infiltrate of AD patients, CD45RO+ CLA+ lymphocytes and inflammatory cells express high levels of IL-31 mRNA and protein [7], [9], [10]. IL-31 signaled via a heterodimeric receptor composed of IL-31RA and oncostatin M receptor β (OSMR-β) [11], [12], which expressed on inflammatory cells, epithelial cells, epidermal keratinocytes and fibroblasts [2], [10], [13]–[15]. Our previous findings revealed that IL-31 could significantly induce the release of pro-inflammatory cytokines IL-1β, IL-6 and AD-related chemokines CXCL1, CXCL8, CCL2 and CCL18 from eosinophils and such induction was further enhanced upon the co-culture of eosinophils and epidermal keratinocytes, via intracellular mitogen activated protein kinases (MAPK), nuclear factor-κB (NF-κB) and phosphatidylinositol 3-kinase (PI3K)-Akt pathways [15]. Therefore, eosinophils and epidermal keratinocytes responded to IL-31 stimulation and were likely to be involved in the dermatitis and pruritis of transgenic mice overexpressing IL-31.

Interleukin (IL)-33 is a member of IL-1 family including IL-1β and IL-18 [16]. Different from the mechanisms of caspase 1-mediated maturation and release of IL-1β and IL-18, full length IL-33 can be released through cell necrosis rather than active secretion [17]. The IL-33 receptor, consisting of ST2 and IL-1 receptor accessory protein, is also widely expressed on Th2 cells, mast cells and eosinophils [17], [18]. Administration of IL-33 in mice not only initiate production of IgE and Th2 cytokines IL-5 and IL-13, but also induce pathological changes including blood and bronchoalveolar lavage fluid eosinophilia, airway hyperresponsiveness, epithelial cell hyperplasia and hypertrophy [16], [19]. IL-33 can be secreted from damaged and inflamed tissues, including endothelial or epithelial cells via scratching behavior, of AD patients [20]. Therefore, IL-33 is proposed to function as an alarmin in sensing damage in various inflammatory diseases including AD [20]. Together with the results from mouse model [16], [19], IL-33 can play a pivotal role in the exacerbation of inflammation in allergic diseases mediated by the activation of eosinophils and basophils [18], [21]. IL-33 could signal via its receptor ST2 with the activation of downstream signaling molecules including NF-κB and MAPK [16], [21]. Our previous studies also demonstrated that IL-33-mediated survival enhancement, induction of adhesion molecules, and release of cytokines and chemokines of eosinophils were differentially regulated by the activation of NF-kB, p38 MAPK and ERK pathways [18].

The histology of AD is characterized by a dermal and epidermal inflammatory infiltrate including eosinophils [6], [22]. Eosinophil infiltration and activation have been shown in AD skin lesions [23], [24]. Both tissue and blood eosinophilia are features in acute and chronic stages of AD and they were found to positively correlate with disease severity [25], [26]. Moreover, eosinophilic granular protein such as eosinophil derived neurotoxin deposition has been found in nearly all biopsies of AD lesions [27], [28]. The induction of eosinophil chemokine eotaxin in dermal fibroblasts only occurs upon the stimulation with Th2 cytokines IL-4 and IL-13 [29]. Moreover, CCL5 and eotaxin can mediate the infiltration of eosinphils into the inner dermal fibroblast layer causing inflammation of AD [29]–[31]. Therefore, we hypothesize that the induction of pluritogenic cytokine IL-31 and alarmin IL-33 may subsequently together activate the infiltrating eosinophils interacting with dermal fibroblasts for allergic inflammation in AD. In an attempt to further evaluate the immunopatholgical role of IL-31 and IL-33 in AD, the underlying intracellular mechanisms of the co-culture of eosinophils and dermal fibroblasts was investigated in the present *in vitro* experimental study.

## Materials and Methods

### Materials

Recombinant human IL-31 and IL-33 were purchased from R&D Systems, Minneapolis, MN, USA. Inhibitor (I)κB-α phosphorylation inhibitor BAY11-7082, extracellular signal-regulated kinase (ERK) inhibitor U0126, c-Jun N-terminal kinase (JNK) inhibitor SP600125, p38 MAPK inhibitor SB203580 and PI3K inhibitor LY294002 were purchased from Calbiochem Corp, San Diego, CA, USA. SB203580 was dissolved in water while U0126, LY294002, SP600125, and BAY11-7082 and calcitriol were dissolved in dimethyl sulfoxide (DMSO). In all studies, the final concentration of DMSO was 0.1% (vol/vol).

### Endotoxin-free solutions

Cell culture medium was purchased from Gibco Invitrogen Corp, Carlsbad, CA, USA, free of detectable lipopolysaccharide (LPS, <0.1 EU/ml). All other solutions were prepared using pyrogen-free water and sterile polypropylene plasticware. No solution contained detectable LPS, as determined by the Limulus amoebocyte lysate assay (sensitivity limit 12 pg/ml; Biowhittaker Inc, Walkersville, MD, USA).

### Isolation of human blood eosinophils from buffy coat and eosinophil culture

Fresh human buffy coat obtained from the healthy volunteers of Hong Kong Red Cross Blood Transfusion Service was diluted 1∶2 with PBS at 4°C and centrifuged using an isotonic Percoll solution (density 1.082 g/ml; Amersham and Pharmacia Biotech, Uppsala, Sweden) for 30 min at 1,000×*g*. The eosinophil-rich granulocyte fraction was collected and washed twice with cold PBS containing 2% fetal bovine serum (FBS) (Gibco). The cells were then incubated with anti-CD16 magnetic beads (Miltenyi Biotec, Bergisch Gladbach, Germany) at 4°C for 45 min and CD16-positive cells were depleted by passing through a LS+ column (Miltenyi Biotec) within a magnetic field. With this preparation, the drop-through fraction contained eosinophils with a purity of at least 99% as assessed by Hemacolor rapid blood smear stain (E Merck Diagnostica, Darmstadt, Germany). The isolated eosinophils were cultured in RPMI 1640 medium (Gibco) supplemented with 10% FBS and 20 mM Hepes (Gibco). The above protocol using human eosinophils purified from human buffy coat was approved by the Clinical Research Ethics Committee of The Chinese University of Hong Kong-New Territories East Cluster Hospitals.

### Co-culture of eosinophils and dermal fibroblasts

The human primary dermal fibroblasts were purchased from Invitrogen Corp., Carlsbad, CA, USA. Dermal fibroblasts were grown in medium 106 with low serum growth supplement (Invitrogen) in cell culture flasks at 37°C with 5% CO_2_, 95% humidified air until confluence to cell monolayer. The medium was then replaced with RPMI 1640 medium supplemented with 10% FBS without or with eosinophils (fibroblasts/eosinophils ratio of 1∶3).

### Co-culture of fixed eosinophils and fibroblasts

Confluent fibroblasts or eosinophils were treated with 1% paraformaldehyde in PBS on ice for 1 h to prevent the release of mediators from cells while preserving the cell membrane integrity to maintain intercellular interaction. After fixation, cells were washed at least 10 times with PBS containing 2% FBS, and fixed or unfixed eosinophils and fibroblasts were co-cultured in RPMI 1640 supplemented with 10% FBS.

### Co-culture of eosinophils and fibroblasts in the presence of transwell inserts

To prevent direct interaction between eosinophils and fibroblasts in the co-culture, transwell inserts (pore size: 0.4 µM) (BD Biosciences Corp, San Jose, CA, USA) were used to separate these two cells into two compartments. Confluent fibroblasts and eosinophils were cultured together in the presence of transwell inserts, in which eosinophils and fibroblasts were placed in the upper and lower compartment, respectively.

### Quantitative analysis of IL-6, CXCL1, CXCL10, CCL2, CCL5 and CXCL8

Concentrations of pro-inflammatory cytokine IL-6 and chemokine CXCL1 in culture supernatant were quantitated with Bio-plex pro assay using Luminex Bio-plex 200 suspension array system (Bio-Rad Corp., Hercules, CA, USA). Chemokines CXCL10, CCL2, CCL5 and CXCL8 were quantitated with human chemokine cytometric bead array (CBA) kit (BD Pharmingen Corp, San Diego, CA, USA) using 4-color FACSCalibur flow cytometer (BD Biosciences Corp., San Jose, CA, USA).

### Western blot analysis

Cells were washed with ice-cold PBS, and lysed in 0.2 ml lysis buffer (20 mM Tris-HCl, pH 8.0, 120 mM NaCl, 1% Triton X-100, 10 mM EDTA, 1 mM EGTA, 0.05% 2-mercaptoethanol, 1× protease inhibitors). Cell debris was removed by centrifugation at 14,000 *g* for 15 min, and the supernatant was boiled in Laemmli sample buffer (Bio-Rad) for 5 min. An equal amount of proteins was subjected to sodium dodecyl sulfate-10% polyacrylamide gel electrophoresis before blotting onto a PVDF membrane (GE Healthcare Bio-Sciences Corp, Piscataway, NY, USA). The membrane was blocked with 5% skimmed milk in Tris-buffered saline with 0.05% Tween 20, pH 7.6 for 1 h at room temperature, and probed with primary rabbit anti-human β-actin (R & D Systems, Minneapolis, MN, USA), rabbit anti-human OSMR-β (Santa Cruz Biotechnology Inc, Santa Cruz, CA, USA), goat anti-human IL-31RA (R & D Systems), or mouse anti-human ST2 antibodies (R & D Systems) (Lifespan Biosciences Inc., Seattle, WA , USA) at 4°C overnight. After washing, membrane was incubated with secondary goat anti-rabbit antibody or sheep anti-mouse antibodies coupled to horseradish peroxidase (GE Healthcare) for 1 h at room temperature. Antibody-antigen complexes were then detected using enhanced chemiluminescence detection system (GE Healthcare) [18].

### Immunofluorescence staining and flow cytometry

To determine the expression of adhesion molecule and ST2 on the cell surface, the cells were washed and resuspended with cold PBS after preceding treatments. After blocking with 2% human pooled serum for 20 min at 4°C and washing with cold PBS, cells were incubated with mouse anti-human intercellular adhesion molecule (ICAM)-1-FITC conjugate (BD Pharmingen), mouse anti-human ST2-FITC conjugate (MD Biosciences GmbH, Switzerland), or mouse IgG_1_ isotypic control antibody (BD Pharmingen) for 30 min at 4°C in the dark. After washing, cells were resuspended in 1% paraformaldehyde as fixative and subjected to flow cytometric analysis.

To determine the intracellular expression of phosphorylated signaling molecules, cells were fixed with 4% paraformaldehyde for 10 min at 37°C after preceding treatments. After centrifugation, cells were permeabilized in ice-cold methanol for 30 min and then stained with FITC-conjugated mouse anti-human phosphorylated Akt, FITC-conjugated phosphorylated ERK1/2, FITC-conjugated phosphorylated JNK, FITC-conjugated phosphorylated p38 MAPK, FITC-conjugated phosphorylated IκB-α, rabbit anti-human phospho-STAT3 (Tyr705) antibody (Cell Signaling Technology Inc., Bervely, MA, USA) together with FITC goat anti-rabbit antibody (BD Pharmingen), or corresponding mouse or rabbit IgG_1_ isotypic antibody (BD Pharmingen) for 30 min at 4°C in dark. Cells were then washed, resuspended and subjected to analysis. Expression of surface adhesion molecules and intracellular phosphorylated signaling molecules of 10,000 viable cells was analyzed by flow cytometry (FACSCalibur) as arithmetic mean of mean fluorescence intensity (MFI) plus SD of three independent experiments with the subtraction with appropriate isotypic control.

### Statistical analysis

All data were expressed as mean ± SD from three independent experiments. The statistical significance of differences was determined by one-way ANOVA or unpaired t-test. Any difference with p-values<0.05 was considered significant. When ANOVA or unpaired t-test indicated a significant difference, Bonferroni *post hoc* test was then used to assess the difference between groups. All analysis was performed using the Statistical Package for the Social Sciences (SPSS) statistical software for Windows, version 16.0 (SPSS Inc, Chicago, IL, USA).

## Results

### Surface expression of receptors for IL-31 and IL-33 on human eosinophils and dermal fibroblasts

**Figure 1:**
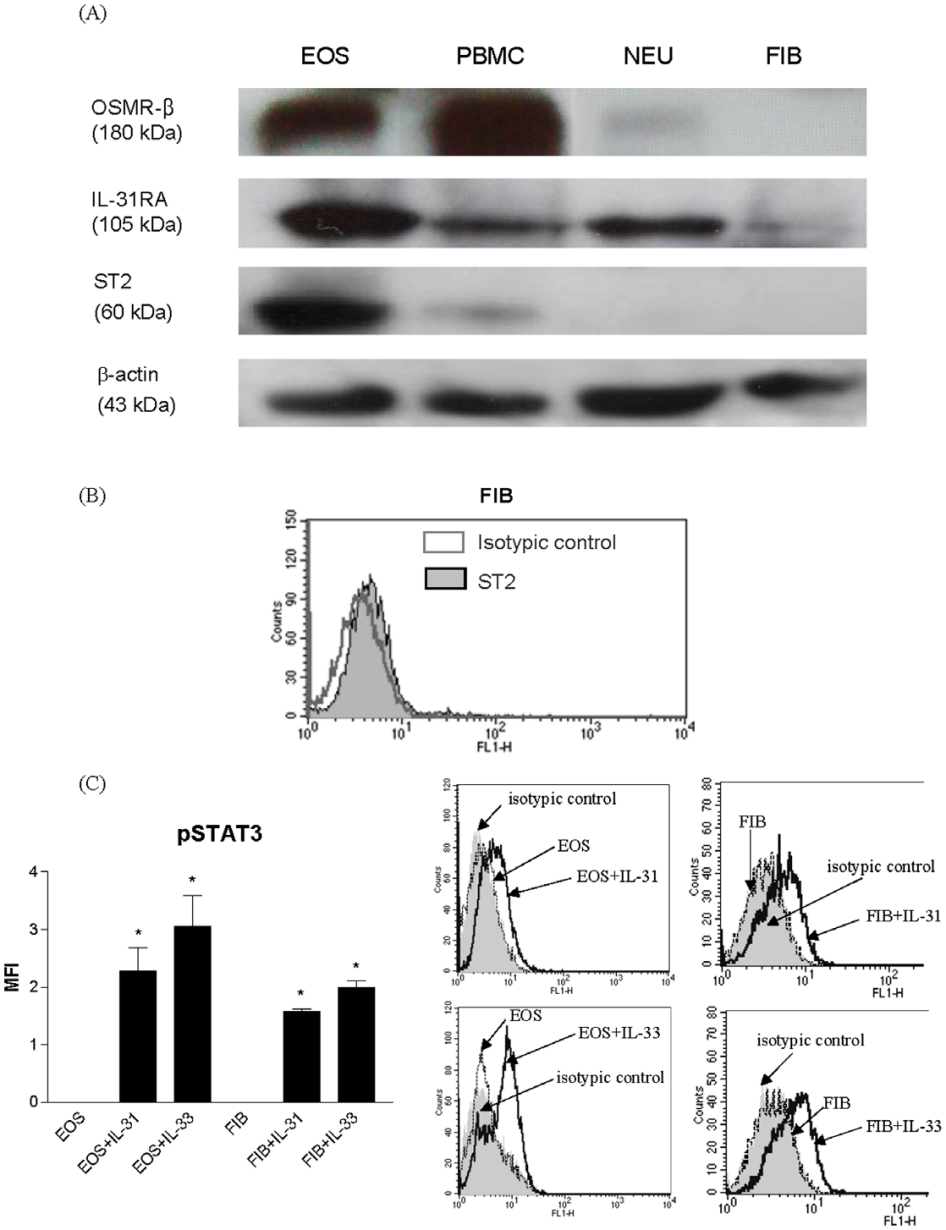
Surface expression of receptors for IL-31 and IL-33 on human eosinophils and fibroblasts. (A) Surface expression of OSMR-β, IL-31RA and ST2 on eosinophils, peripheral blood mononuclear cells (PBMC), neutrophils and fibroblasts (5×10^5^ cells) was determined Western blot. Triplicate experiments were performed with essentially identical results and representative figure is shown. Neutrophils and PBMC were served as cell controls, and β-actin was used as protein control to ensure an equal amount of loaded protein. (B) Surface expression of ST2 on fibroblasts was determined by flow cytometry. Results are expressed as representative histogram of relative cell counts with mean fluorescence intensity (MFI). (C) Functional activity of the receptor complex for IL-31 and IL-33. IL-31 or IL-33 (50 ng/ml) was added to eosinophils or fibrolasts (5×10^5^ cells) for 10 min. Phosphorylation of STAT3 (pSTAT3) in eosinophils and fibroblasts was determined by flow cytometry. Results are shown in MFI subtracting corresponding isotypic control and expressed as the arithmetic mean plus SD of three independent experiments in bar chart. Representative histograms illustrate the intracellular expression of pSTAT3 upon IL-31 or IL-33 stimulation in permeabilized eosinophils and fibroblasts. *p<0.05 comparing with corresponding medium control. EOS: eosinophils; NEU: neutrophils; FIB: dermal fibroblasts.

IL-31 and IL-33 signal through a heterodimeric receptor composed of IL-31RA and OSMR-β, and ST2, respectively [11], [12], [16]. As shown in Figure 1A and 1B, IL-31RA, OSMR-β, and ST2 were constitutively expressed on the surface of human eosinophils while IL-31RA and ST2 were found to be expressed on dermal fibroblasts. Since the antibody against ST2 might be optimized for flow cytometry but not Western blot, we confirmed the ST2 expression on fibroblasts using flow cytometry but not Western blot (Figure 1A and 1B).

Previous studies have demonstrated that the binding of IL-31 and IL-33 to its receptor complex could induce the phosphorylation of STAT3, which exerted a dominant function in the entire receptor complex [11], [32], [33]. In order to show that the IL-31 and IL-33 receptors on both cell types are functionally active, phosphorylation of STAT3 in eosinophils and firoblasts upon IL-31 and IL-33 stimulation was examined. Results in Figure 1C illustrates that upon IL-31 and IL-33 treatment, phosphorylation of STAT3 was significantly increased in both fibroblasts and eosinophils, indicating that the IL-31 and IL-33 receptors expressed on both cell types were functional active.

### Cytokine and chemokine release upon the interaction of eosinophils and dermal fibroblasts activated by IL-31 and IL-33

**Figure 2:**
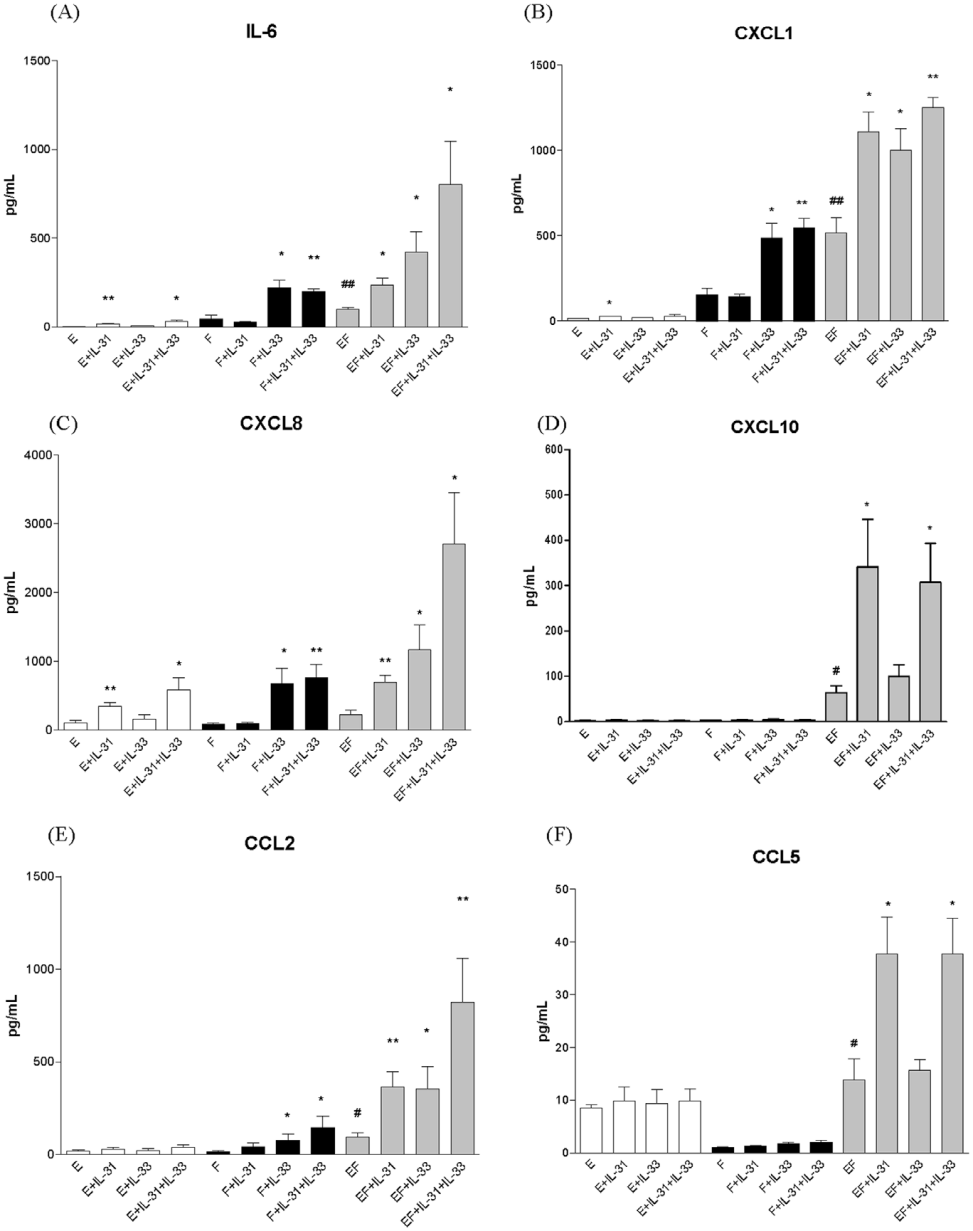
Effect of IL-31 and IL-33 on the induction of IL-6, CXCL1, CXCL8, CXCL10, CCL2 and CCL5 upon the interaction of eosinophils and fibroblasts. Eosinophils (3×10^5^ cells) and confluent fibroblasts (1×10^5^ cells) were cultured either together or separately with or without IL-31 and/or IL-33 (50 ng/ml) for 24 h. Cytokines and chemokines released in culture supernatant were determined by either CBA or Bio-plex pro assay. Results are expressed as the arithmetic mean plus SD of three independent experiments. E: eosinophils; F: dermal fibroblasts; EF: co-culture of eosinophils and dermal fibroblasts *p<0.05, **p<0.01 when compared between treatment group and control cell group; #p<0.05, ##p<0.01 when compared between co-culture group and single cell group.

Figure 2 shows the cytokine and chemokine release when eosinophils and fibroblasts were cultured either together or separately with or without IL-31 and IL-33 treatment. Co-culture of eosinophils and fibroblasts significantly increased the release of pro-inflammatory cytokine IL-6 and AD-related chemokines CXCL1, CXCL10, CCL2 and CCL5, while comparing with those of eosinophils alone or fibroblast alone. Such increases were further enhanced with IL-31 and IL-33 stimulation. IL-31 and IL-33 could significantly induce the release of CXCL8 from eosinophils and fibroblasts, respectively, which was further enhanced upon the co-culture of eosinophils and fibroblasts with the stimulation by IL-31 and IL-33 (Figure 2).

### Source of the release of cytokines and chemokines in the co-culture system upon IL-31 and IL-33 stimulation

**Figure 3:**
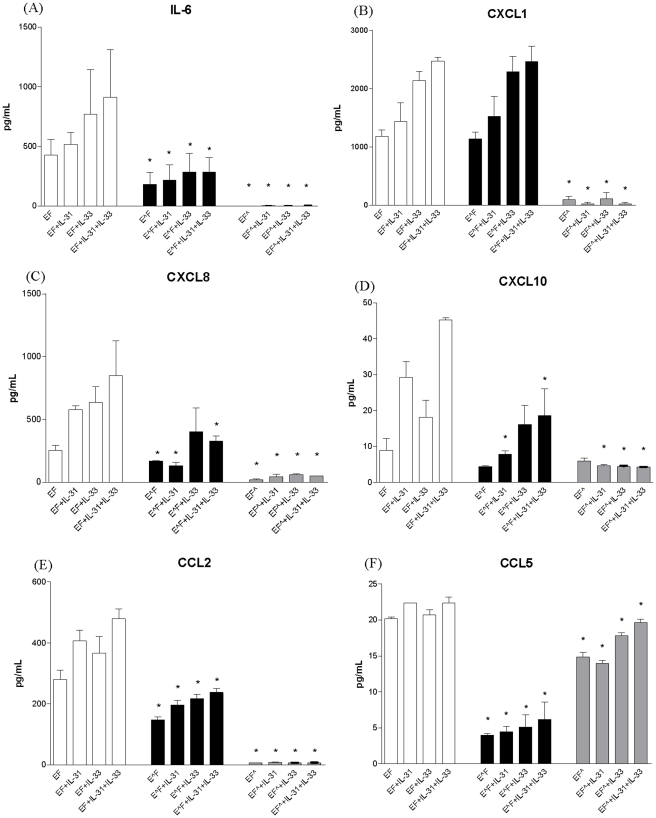
Source of IL-6, CXCL1, CXCL8, CXCL10, CCL2 and CCL5 in co-culture of eosinophils and fibroblasts under IL-31 and IL-33 stimulation. Eosinophils (3×10^5^ cells) and confluent fibroblasts (1×10^5^ cells) were treated with or without 1% paraformaldehyde for 1 h on ice prior to being cultured together with or without IL-31 and/or IL-33 (50 ng/ml) for 24 h. Cytokines and chemokines released in culture supernatant were determined by either CBA or Bio-plex pro assay. Results are expressed as the arithmetic mean plus SD of three independent experiments. E: unfixed eosinophils; E∧: fixed eosinophils; F: unfixed dermal fibroblasts; F∧: fixed dermal fibroblasts *p<0.05 when compared with corresponding unfixed control.

To investigate the source of cytokines and chemokines released in the co-culture supernatant, 1% paraformaldehype was used to fix either eosinophils or fibroblasts to prevent cytokine and chemokine release while preserving the cell membrane integrity to maintain intercellular interaction. We compared the cytokine and chemokine levels in the co-culture of normal unfixed cells with the cells fixed with 1% paraformaldehyde (Figure 3). The fixation of fibroblasts alone but not eosinophils could completely suppress the release of IL-6, CCL2, CXCL1, CXCL8 and CXCL10, with or without IL-31 and IL-33 stimulation, thereby indicating that fibroblasts were the major cells contributed to the production of IL-6, CCL2, CXCL1, CXCL8 and CXCL10 in the co-culture system (Figure 3A–E). In the co-culture of fixed eosinophils and unfixed fibroblasts, the stimulatory effect of the co-culture on the release of CCL5 without or with IL-31 and IL-33 stimulation were greatly suppressed (Figure 3F). On the contrary, fixation of fibroblasts alone could only partially suppress the secretion of CCL5 in co-culture with or without IL-31 and IL-33 stimulation, indicating that eosinophils were the main source for releasing CCL5 in co-culture upon IL-31 and IL-33 stimulation (Figure 3F).

### Effect of transwell inserts on cytokine and chemokine release in IL-31 and IL-33-treated co-culture

**Figure 4:**
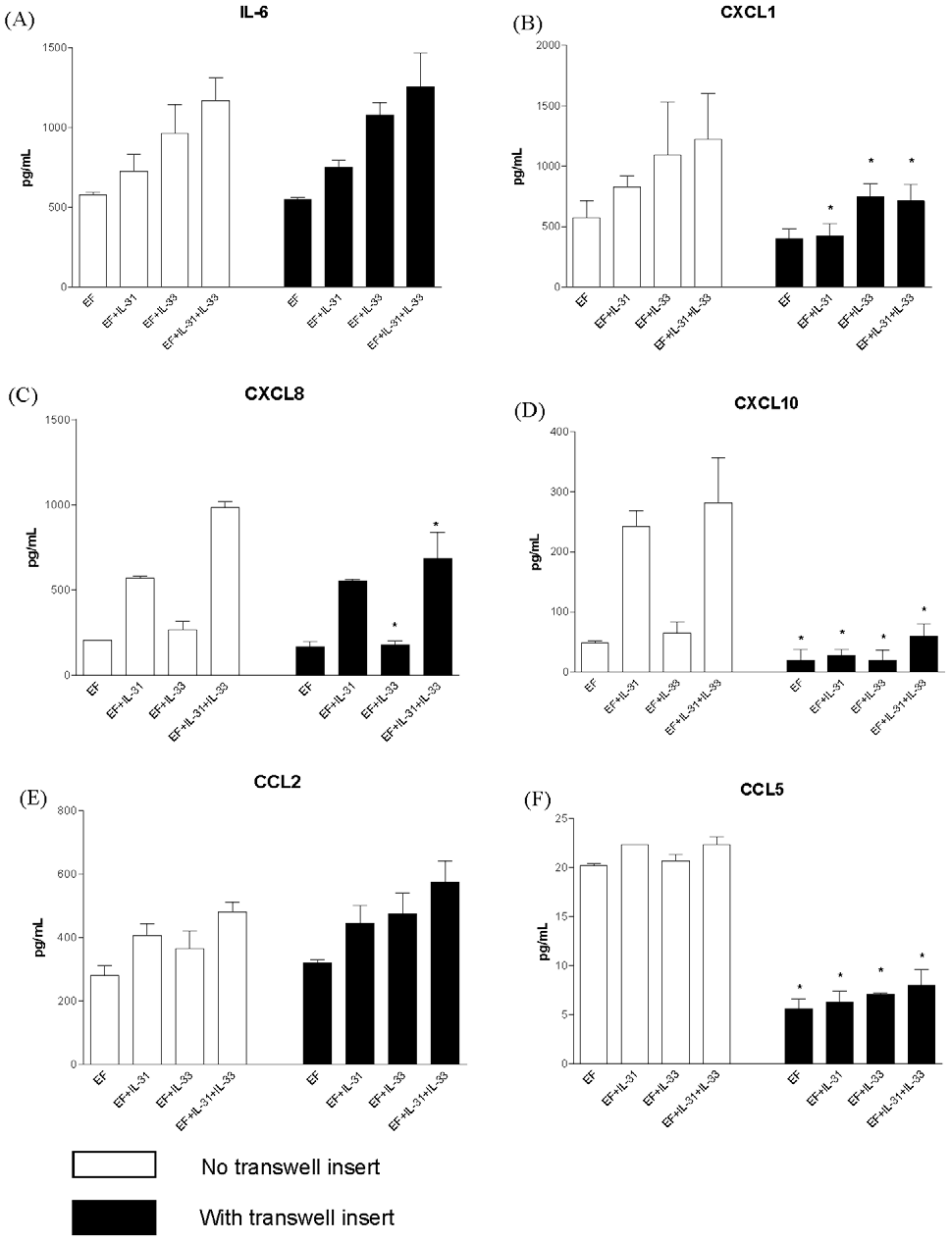
Effect of transwell inserts on the induction of IL-6, CXCL1, CXCL8, CXCL10, CCL2 and CCL5 in co-culture of eosinophils and fibroblasts under IL-31 and IL-33 stimulation. Eosinophils (3×10^5^ cells) and confluent fibroblasts (1×10^5^ cells) were cultured together with or without IL-31 and/or IL-33 (50 ng/ml) in the presence or absence of transwell inserts for 24 h. Cytokines and chemokines released in culture supernatant were determined by either CBA or Bio-plex pro assay. Results are expressed as the arithmetic mean plus SD of three independent experiments. EF: co-culture of eosinophils and dermal fibroblasts *p<0.05 when compared with corresponding control without transwell insert.

To examine whether direct interaction was essential for IL-6, CCL2, CCL5, CXCL1, CXCL8 and CXCL10 release in the co-culture upon IL-31 and IL-33 stimulation, transwell inserts (pore size: 0.4 µM) were used to separate eosinophils and fibroblasts into two compartments in the co-culture system. Figure 4B, 4D, and 4F shows that the presence of transwell inserts significantly suppress the secretion of CXCL1, CXCL10 and CCL5 in co-culture with or without IL-31 and IL-33 stimulation while transwell inserts could significantly suppress the IL-33-inuced CXCL8 release from co-culture (Figure 4C). These implied that the release of these cytokines and chemokines in co-culture might depend on direct interaction between eosinophils and dermal fibroblasts. However, the transwell inserts did not exhibit any significant effect on the release of IL-6 and CCL2 release from co-culture with or without IL-31 and IL-33 stimulation, thereby indicating soluble mediators or cytokines rather than direct interaction may be responsible for the synergic induction of IL-6 and CCL2 from co-culture (Figure 4A and 4E).

### Effect of IL-31 and IL-33 on adhesion molecule expression on eosinophils and dermal fibroblast in co-culture system

**Figure 5:**
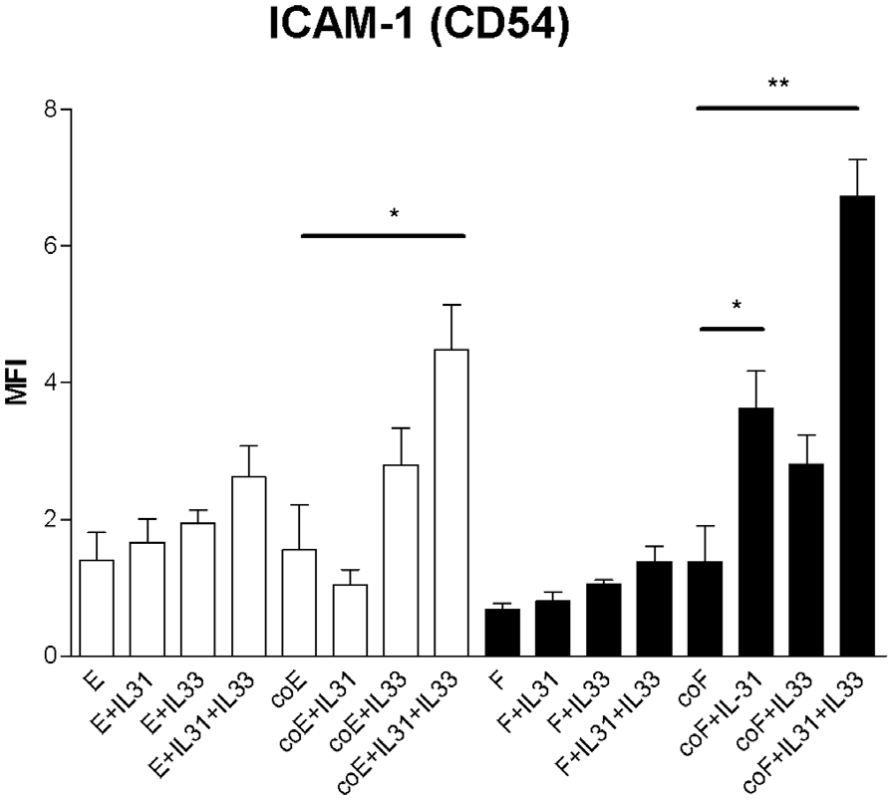
Effect of IL-31 and IL-33 on the surface expression of ICAM-1 on eosinophils and fibroblasts in the co-culture. Eosinophils (3×10^5^ cells) and confluent fibroblasts (1×10^5^ cells) were cultured either together or separately with or without IL-31 and/or IL-33 (50 ng/ml) for 16 h. Surface expression of ICAM-1 on 10,000 cells was analyzed by flow cytometry as MFI. Results have been normalized by subtracting appropriate isotypic control and expressed as the arithmetic mean plus SD of three independent experiments. E: eosinophils only; coE: eosinophils in co-culture; F: dermal fibroblasts; coF: fibroblasts in co-culture *p<0.05, **p<0.01 when compared between groups denoted by horizontal lines.

As shown in Figure 5, combined treatment of IL-31 and IL-33 could significantly up-regulate the surface expression of ICAM-1 on eosinophils in co-culture while IL-31 could significantly up-regulate the expression of ICAM-1 on fibroblasts in co-culture with or without IL-33 treatment. As we observed that all eosinophils and fibroblasts expressed ICAM-1, the increase in MFI was due to the upregulation of ICAM-1 on cell surface rather than the increase of the number and % of ICAM-1 positive cells.

### Intracellular signaling pathways involved in the interaction of eosinophils and dermal fibroblasts under IL-31 and IL-33 stimulation

**Figure 6:**
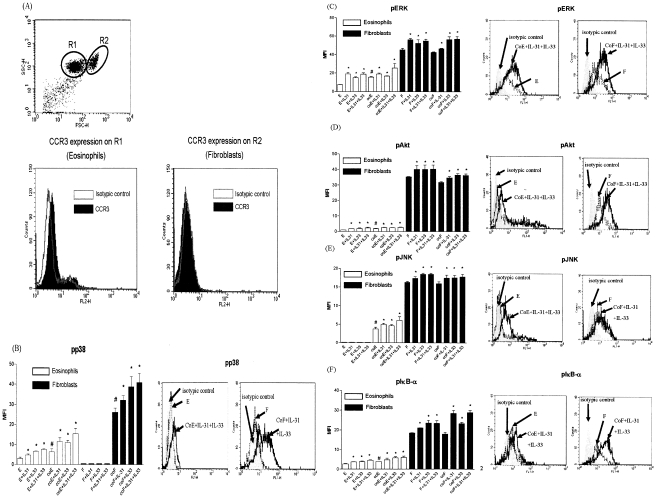
Activation of ERK, p38 MAPK, Akt, JNK and IκB-α in co-culture of eosinophils and fibroblasts under IL-31 and IL-33 stimulation. Eosinophils (3×10^5^ cells) and confluent fibroblasts (1×10^5^ cells) were cultured either together or separately with or without IL-31 and/or IL-33 (50 ng/ml) stimulation for 10 min. (A) After fixation and permeabilization, eosinophils (3×10^5^ cells) and fibroblasts (1×10^5^ cells) formed discrete populations and were gated based on forward and side light scatter with CCR3 staining to distinguish eosinophils (R1) and fibroblasts (R2). The intracellular contents of phosphorylated (B) p38 MAPK, (C) ERK, (D) Akt, (E) JNK and (F) IκB-α of permeabilized eosinophils and fibroblasts were measured by intracellular immunofluorescence staining using flow cytometry. Results are shown in MFI subtracting corresponding isotypic control and expressed as the arithmetic mean plus SD of three independent experiments in bar charts. Representative histograms illustrate the intracellular expression of phosphorylated (B) p38 MAPK, (C) ERK, (D) Akt, (E) JNK and (F) IκB-α of permeabilized eosinophils and fibroblasts. E: eosinophils only; coE: eosinophils in co-culture; F: dermal fibroblasts only; coF: dermal fibroblasts in co-culture *p<0.05 when compared between treatment group and control cell group, #p<0.05 when compared between co-culture group and single cell group.

**Figure 7:**
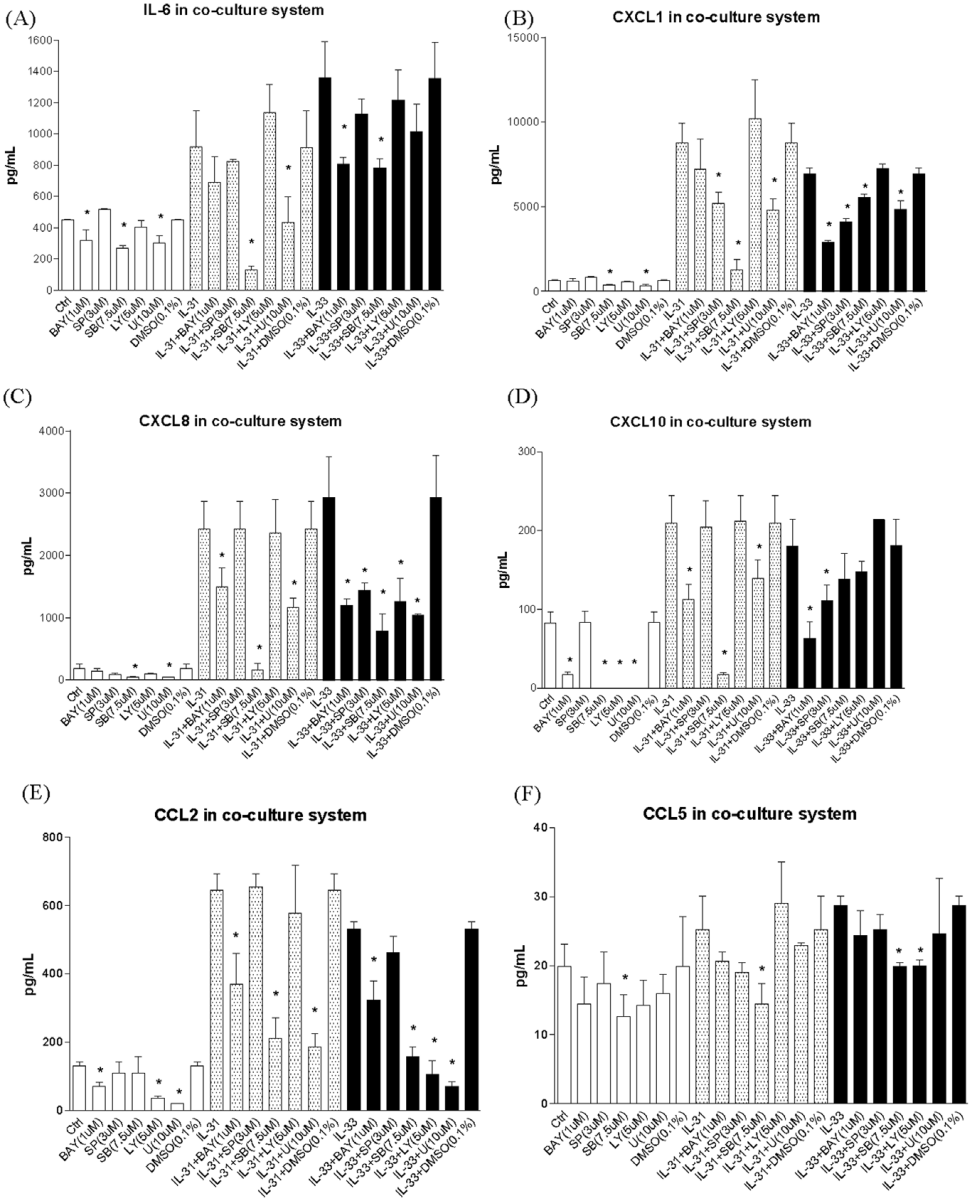
Effects of signaling molecule inhibitors on the release of IL-6, CXCL1, CXCL8, CXCL10, CCL2 and CCL5 from co-culture of eosinophils and fibroblasts with or without IL-31 and IL-33 stimulation. Eosinophils (3×10^5^ cells) cultured together with confluent fibroblasts (1×10^5^ cells) were pretreated with BAY11-7082 (1 µM), LY294002 (5 µM), U0126 (10 µM), SP600125 (3 µM) or SB203580 (7.5 µM) for 45 min, followed by incubation with or without IL-31 or IL-33 (50 ng/ml) in the presence of inhibitors for further 24 h. Released cytokines and chemokines in culture supernatant were determined by either CBA or Bio-plex pro assay. Results are expressed as the arithmetic mean plus SD of three independent experiments. DMSO (0.1%) was used as the vehicle control. Ctrl: medium control; BAY: BAY11-7082, LY: LY294002, U: U0126, SB: SB203580, SP: SP600125 *p<0.05 when compared between treatment group and control cell group.

**Figure 8:**
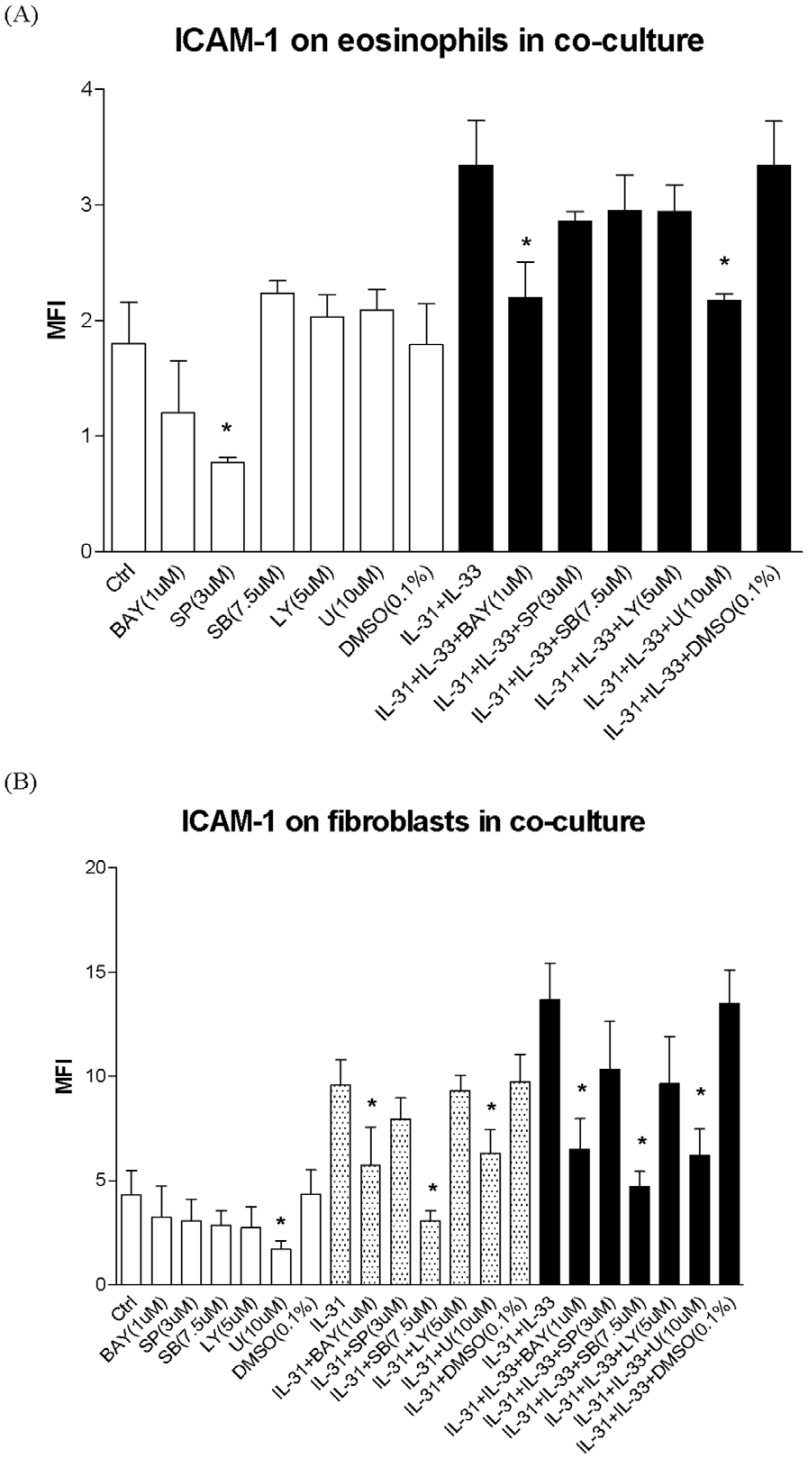
Effects of signaling molecule inhibitors on the cell surface expression of ICAM-1 on eosinophils or fibroblasts in the co-culture. Eosinophils (3×10^5^ cells) and confluent fibroblasts (1×10^5^ cells) cultured together, were pretreated with BAY11-7082 (1 µM), LY294002 (5 µM), U0126 (10 µM), SP600125 (3 µM) or SB203580 (7.5 µM) for 45 min, followed by incubation with or without IL-31 or IL-33 (50 ng/ml) in the presence of inhibitors for further 16 h. Surface expression of ICAM-1 on 10,000 cells was analyzed by flow cytometry as MFI. Results have been normalized by subtracting appropriate isotypic control and expressed as the arithmetic mean plus SD of three independent experiments. DMSO (0.1%) was used as the vehicle control. Ctrl: medium control; BAY: BAY11-7082, LY: LY294002, U: U0126, SB: SB203580, SP: SP600125 *p<0.05 when compared between treatment group and control cell group.

To investigate the underlying signaling mechanisms, intracellular staining by quantitative flow cytometry was employed. After fixation and permeabilization, eosinophils and fibroblasts formed discrete populations and were gated on the basis of their forward and side light scatter (Figure 6A). In order to distinguish these two populations, cells were stained with anti-human CCR3 antibody because CCR3 is an exclusive chemokine receptor expressed on eosinophils, but not on fibroblasts. Figure 6A indicates that the gated population with lower forward side scatter (FSC), R1, showing positive CCR3 expression is eosinophils; while the one with higher FSC, R2, showing no CCR3 expression is fibroblasts. Figure 6B–F show that upon the stimulation by IL-31, IL-33 and co-culture, intracellular ERK, p38 MAPK, Akt, JNK and IκB-α were differentially phosphorylated in eosinophils and fibroblasts. In Figure 6B, both IL-31 and IL-33 could significantly activate p38 MAPK of fibroblast only in co-culture but not in fibroblast alone. In Figure 6C–F, IL-31 and IL-33 exhibited significant higher activation of ERK, Akt, JNK and NF-κB in fibroblasts with or without co-culture than that of eosinophils (all p<0.05). To further verify the involvement of the above signaling pathways in the interaction between eosinophils and fibroblasts upon IL-31 and IL-33 stimulation, various signaling molecule inhibitors were used. As shown in Figures 7 and 8, PI3K inhibitor LY294002 (5 µM), ERK inhibitor U0126 (10 µM), JNK inhibitor SP600125 (3 µM), p38 MAPK inhibitor SB203580 (7.5 µM) and NF-κB inhibitor BAY11-7082 (1 µM) could differentially suppress the IL-31 and IL-33-induced release of cytokines and chemokines, and also the expression of ICAM-1 on eosinophils and fibroblasts (Figures 7 and 8). SB203580 and U0126 could significantly inhibit IL-31 stimulated IL-6, CXCL8, CCL2, CXCL10 and CXCL1 release from co-culture (all p<0.05, Figure 7A–E) while only SB203580 could significantly inhibit CCL5 release from IL-31 activated co-culture (all p<0.05, Figure 7F). BAY11-7082 and SB203580 could significantly inhibit IL-6, CXCL1, CXCL8 and CCL2 release from IL-33 activated co-culture (all p<0.05, Figure 7A, 7B, 7C, and 7E). BAY11-7082 and SP600125, and SB203580 and LY294002 could significantly inhibit IL-33 stimulated CXCL10 and CCL5 release from co-culture, respectively (all p<0.05, Figure 7D and 7F).

As shown in Figure 8A, BAY11-7082 and U0126 could significantly suppress IL-31 and IL-33 combined effect on ICAM-1 expression on eosinophils in co-culture (both p<0.05). BAY11-7082, SB203580 and U0126 could significantly suppress IL-31 induced ICAM-1 expression on fibroblasts while BAY11-7082, SB203580 and U0126 could significantly suppress IL-31 and IL-33 combined effect on the ICAM-1 induction on fibroblast in co-culture (all p<0.05, Figure 8B). Solvent control DMSO (0.1%) did not have any significant effect on the release of cytokines and chemokines and expression of ICAM-1.

## Discussion

Although the presence of eosinophils in the inflammatory infiltrate of AD has long been recognized [22]–[26], their pathogenic role in the development of local inflammation of the disease is not well understood. Eosinophil infiltration into the inner dermal fibroblast layer causing inflammation in AD has been well established [29]–[31]. Investigation of the interaction between eosinophils and fibroblasts may therefore help to elucidate the mechanism of initiating local inflammatory response in AD. Recently, there has been mounting evidence suggesting an important role of IL-31 and IL-33 in the pathogenesis of AD [1], [2], [7], [15], [17]. As shown in Figure 1, we showed that primary human eosinophils constitutively expressed functional receptor complex for IL-31 and IL-33 receptor ST2 which is in concordance with our previous publications [15], [18]. Moreover, IL-31RA and ST2 were found to be expressed on dermal fibroblasts (Figure 1). Consequently, we demonstrated that IL-31 and IL-33 together could stimulate the co-culture of eosinophils and fibroblasts to secrete high levels, up to hundred fold, of pro-inflammatory cytokine IL-6 and AD-related chemokines CXCL1, CXCL8, CXCL10, CCL2 and CCL5 (Figure 2). Furthermore, eosinophils were found to be the main source for releasing eosinophil and basophil-related chemokine CCL5 while fibroblasts were the major source for the production of IL-6, CCL2, CXCL1, CXCL8 and CXCL10 in co-culture upon IL-31 and IL-33 stimulation (Figure 3). AD is a multifactorial disorder characterized with Th2 cells overproducing IL-6, IL-5, IL-4 and IL-13 [22], [34]. IL-6 can activate Th lymphocytes and induce the Th2 immune responses, the synthesis of acute-phase proteins and mediate various inflammatory responses [33]. *Staphylococcus aureus*, the concurrent bacterial infection in AD, can induce IL-6 production from dermal fibroblasts causing skin inflammation [30]. Clinically, IL-6 is released in the cutaneous response to allergen challenge in atopic individuals [35]. In mice model, IL-6 has been shown to be a major cause of a high irritant dermatitis [36]. In fact, a recent clinical trial also revealed that the interruption of IL-6 receptor signaling improves the severity of AD [37]. In view of the above, the induction of IL-6 in the co-culture of eosinophils and fibroblasts upon IL-31 stimulation might therefore play an important role in the development of local inflammatory responses found in AD. For chemokines, CCL2 is known to be AD-associated chemokine to recruit dendritic cell precursors from circulation to the inflammatory sites of atopic skin; while both CXCL8 and CXCL1 are potent chemoattractants for neutrophils and basophils [38]–[40]. CXCL10 and CCL5 are chemokines for activated T cells, and eosinophils and basophils, respectively [41]. Therefore, pruritogenic cytokine IL-31 and alarmin IL-33 can together activate the infiltrating eosinophils, basophils and other immune effector cells interacting with dermal fibroblasts for allergic inflammation in AD. Actually, we also observe IL-33 alone could induce the release of AD-related chemokines from co-culture of eiosinophils and epidermal keratinocytes, and IL-31 and IL-33 could synergistically stimulate AD-related chemokines release from basophils interacting with fibroblasts (unpublished data).

Results of transwell experiments showed that the release of CXCL1, CXCL8, CXCL10, CCL2 and CCL5 were dependent, at least partly, on the direct interaction between eosinophils and fibroblasts. One possible mechanism for the stimulatory effect on the induction of the above cytokine and chemokines might be the direct activation of cells through the interaction between their surface adhesion molecules such as ICAM-1. Following the endothelium-regulated processes of rolling, tethering, adhesion and extravasation, eosinophils are attracted to the skin cells by chemoattractants and maintained by adhesion molecules [42]. ICAM-1 is a crucial adhesion molecule present on fibroblast and plays essential role in cell activation and adherence [43]. ICAM-1 interacts with the highest affinity to the integrin family member LFA-1 (CD11a/CD18) [44], [45]. We found that ICAM-1 on both fibroblasts and eosinophils were significantly enhanced in co-culture activated by IL-31 and IL-33 (Figure 5). Such ICAM-1 interaction might provide a potential stimulation on the release of cytokines and chemokines in the co-culture system activated by IL-31 and IL-33.

Regarding the signal transduction mechanism, we used a rapid and quantitative method, intracellular staining using flow cytometry, to investigate the phosphorylation levels of signaling molecules in permeabilized eosinophils and fibroblasts in co-culture under IL-31 and IL-33 stimulation. Our results demonstrated that in the co-culture system, PI3K-Akt, ERK, JNK, p38 MAPK and NF-κB pathways were all activated in eosinophils and fibroblasts upon co-culture with the stimulation by IL-31 and IL-33 (Figure 6). Following previous publications [15], [18], we adopted the optimal concentration of BAY11–7082 (1 µM), U0126 (10 µM), LY294002 (5 µM), SP600125 (3 µM), and SB203580 (7.5 µM) with the highest inhibitory effect without any significant cell toxicity, we further elucidated the involvement of above signaling pathways upon the interaction of eosinophils and fibroblasts in co-culture under the stimulation of IL-31 and IL-33 (Figures 7 and 8). Since we have shown that fibroblasts were the major source for the release of AD-related IL-6, CCL2, CXCL1, CXCL8 and CXCL10 in the co-culture system (Figure 3A–E), it is reasonable that both IL-31 and IL-33 exhibited significant higher activation of ERK, Akt, JNK and NF-κB in fibroblasts than that of eosinophils (Figure 6C–F). NF-κB inhibitor BAY11-7082 and ERK inhibitor U0126 could suppress ICAM-1 expression as well as the release of IL-6 and chemokines from co-culture activated by IL-31 and IL-33 (Figures 7 and 8). It also demonstrated that ICAM-1 expression is essential for the IL-6 and chemokines release in co-culture of esoinophils and fibroblasts mediated via the activation of intracellular ERK and NF-κB. The difference between IL-31 and IL-33 for the induction of IL-6 and chemokines should be related to their differential activation of distinct signaling mechanisms in co-culture. For example, IL-31 and IL-33 induced IL-6 from co-culture was found to be related to the activation of p38 MAPK and ERK, and p38 MAPK and NF-κB, respectively (Figure 7A).

Our group has previously demonstrated the involvement of PI3K-Akt, ERK, p38 MAPK and NF-κB pathways in regulating adhesion molecule expression, cytokine and chemokine release of activated eosinophils upon exposure to different stimuli such as leptin, Th17 cytokines, various microbial products and IL-33 [18], [46]–[48]. Besides, we also reported the involvement of MAPK, PI3K-Akt and NF-κB in activated eosinophils in co-culture with epidermal keratinocytes upon stimulation by IL-31 [15]. Therefore, it is logical that the above pathways are commonly involved in IL-31 and IL-33-induced effects on eosinophils cultured with dermal fibroblasts in the present study. Moreover, our findings on signaling mechanism in fibroblasts concurred with some previous studies that showed the involvement of PI3K-Akt, MAPKs and NF-κB pathways in regulating cytokine and chemokine release from keratinocytes [49]–[51].

In conclusion, this is the first report on the immunopathological role of pruritogenic IL-31 in conjunction with alarmin IL-33 upon the interaction of human eosinophils and dermal firboblasts. Our results demonstrated a stimulatory effect induced by co-culture of eosinophils and fibroblasts on cytokine and AD-related chemokine release. The above stimulatory effects could be significantly enhanced by the AD-related IL-31 and IL-33, probably via the ICAM-1 expression and intracellular signalling mechanisms. Together with previous reports on the participation of IL-31 in pruritus and dermatitis in animal models [2], [3] and IL-33 in AD [17], our results provide insight of the pathological roles of IL-31 and IL-33 in the pathogenesis of AD. In order to further elaborate the immunopathological roles of IL-31 and IL-33 in AD, future study for the IL-31 and IL-33 activating effects on skin-resident eosinophils and dermal fibroblasts obtained from AD patients is needed.
